# The impact of climate change on cancer nursing in Palestine

**DOI:** 10.3332/ecancer.2023.ed129

**Published:** 2023-11-09

**Authors:** Jehad Hawaamdah, Matthew Fowler

**Affiliations:** 1 Department of Continuous Nursing Education, Augusta Victoria Hospital, Rabe’a Al A’ddaweya Street, Mount of Olives/East Jerusalem; 2 Department of Oncology, University Hospitals of Derby and Burton NHS Foundation Trust, Uttoxeter Road, Derby, DE22 3NE, UK

**Keywords:** climate change, cancer nursing, Palestine

## Abstract

Cancer is the third leading cause of death in Palestine, with many cancers diagnosed at a late stage. In contrast to the developed world, two thirds of cancer diagnoses occur between the ages of 15 and 64, moreover, 10% of all cancer diagnoses occur in children under the age of 10 (compared to 0.05% of all new cancer diagnoses in the UK).

Cancer nursing as a speciality in Palestine is newly established in the last 5 years; partly helped by the introduction of the Higher Diploma in Cancer and Palliative Care Nursing, and more recently the delivery of the first intake of the Master of Science in Cancer and Palliative Care Nursing at Bethlehem University.

There are many challenges faced by cancer patients and nurses in Palestine; there is only one facility in the West Bank that delivers radiotherapy, 2 PET-CT scanners for the whole of the West Bank, with no PET-CT or radiotherapy facilities in Gaza. There are 2 haematology units in the West Bank that perform autologous stem cell transplants for adults and any haematology patient (adult or child) requiring an allogeneic stem cell/bone marrow transplant has to be referred to neighbouring Israel or Jordan.

Climate change might have both a direct and indirect impact on the growth of cancers and on cancer treatment and oncology nurses. Over the last 150 years the planet has warmed by over one degree Celsius resulting in disastrous consequences for the environment. Nurses make up the largest number of the healthcare workforce and are ideally placed to have a positive impact on the global warming crisis due to their leadership roles as well as their work in health promotion. They equally do a lot to help cancer patients to deal with its effects and often care for patients from marginalised groups. It is important for nurses to take the lead and move immediately to make health systems more resistant to climate change.

## Introduction

This article will explore the role of the cancer nurse in the Occupied Palestinian Territories, with an exploration of the current situation in the context of the global climate change crisis as well as potential actions the cancer nursing workforce can take to improve the lives of cancer patients and the wider cancer nursing workforce.

Climate change refers to a change in the weather that can be linked directly or indirectly to human actions that change the makeup of the atmosphere around the world. This is in addition to natural fluctuations in the weather that have been seen over similar amounts of time [8].

Climate change is most likely to affect cancer control through routes that involve air pollution, being exposed to UV radiation, changes in supplies of water and food, exposure to industrial toxicants, and maybe even infectious diseases that cause cancer [11]. Climate temperature alteration as well as poor air quality, are big and existential health problems that threaten the well-being of the human race, mostly from low and middle income countries due to low capacity for adaptation to such problems (World Health Organization 2021). Climate change can affect health in many ways and take lives or evoke illness due to adverse weather events including heat waves, sand storms and air pollutants: e.g. dengue and malaria, exposure to ultraviolet radiation and skin and lung cancer [9].

These adverse weather events to climate changes will be dangerous for vulnerable groups of people such as children, as well as patients with pre-existing conditions such as cancer [17]. If roads and travel networks are affected by climate events, it might be harder to get to health care services, like regular cancer screening. Some screening immune tests are less effective when the temperature is high [11]. As the number of accidents, infections, and communicable diseases rise after a disaster, the health system may not be able to handle it. This could affect cancer care as resources are moved to help with the disaster [5]. It should never be underestimated how climate change can affect this process, it can change the process of cancer detection, diagnosis and evaluation, The Lancet Commission on pollution and health found that pollution of all kinds are responsible for 43% of lung cancer deaths, and up to 15% of deaths from lung cancer are associated with particle pollution, and the number of mortality caused by particles has increased by 20% in the last 30 years [17].

Palestine is a geographical landscape consisting of two separate areas: The West Bank and Gaza Strip are between a part of Lebanon with a lot of rain and a part of Egypt with little rain. So, the temperature can be warm and dry in the south and warm and wet in the north, the weather in the West Bank and Gaza is mild, with temperatures and amounts of rain that change with altitude. The average summer temperature in the West Bank ranges from 30°C in Jericho to 25°C in Gaza to 22°C in Hebron [16].

This is why the amount of rain in different places varies so much. For instance, the average amount of rain in the middle mountain region is between 400 and 700 millimetres (mm). About 300 mm of rain falls every year on the coastal plains, while about 550 mm falls on the semi-coastal plains [10]. The Jordan Valley is a dry area below sea level that gets about 200 mm of rain a year on average. Due to high levels of radiation, the amount of water that evaporates varies from 1,900 mm per year in hilly areas and the Gaza Strip to 2,600 mm per year in the Jordan Valley. These numbers are very important to consider when planning farming projects or building sewage treatment plants [4].

There are many challenges faced by cancer patients and nurses in Palestine; there is only one facility in the West Bank that delivers radiotherapy, 2 PET-CT scanners for the whole of the West Bank, with no PET-CT or radiotherapy facilities in Gaza. There are 2 hematology units in the West Bank that perform autologous stem cell transplants for adults, and any hematology patient (adult or child) requiring an allogeneic stem cell/bone marrow transplant has to be referred to neighboring Israel or Jordan. The Gaza strip is a fragile state with substantial public health and development challenges. Any patient in the Gaza strip who requires intensive treatment for a haematological diagnosis will be required to obtain a permit to travel to the West Bank for their treatment; this can take weeks to happen sometimes. Cancer treatments and many diagnostic investigations, including PET-CT scans, radiation, Bone marrow transplantation are not available and patients need to travel east to hospitals in West bank and East Jerusalem, often enduring long processes due to securing the necessary travel permits because radiotherapy is only available in east Jerusalem, for example of a patient requiring radiotherapy post breast surgery in Gaza will need a permit to travel to West Bank. Often these women will undergo mastectomies due to lack of availability for radiotherapy in Gaza [4].

The cancer facilities which are treating cancer patients need emergency preparedness for such issues to address the climate changes and plan to avoid the threats by reducing the exposure to environmental hazards. Patients with cancer can get prepared for emergencies by ensuring that they have their medicines and information about their diagnoses and treatments to hand. This means that people with cancer become particularly at risk when natural events affect their ability to get care [10]. As climate change alters the frequency, severity, and behaviour of extreme weather conditions, it makes it harder for communities to prepare for and react to more unpredictable and severe weather. This makes them more vulnerable to natural disasters [14].

Cancer nurses are the most trusted professionals to be in contact with patient and families, identify which patients are at risk, why they are vulnerable and assist them to understand the climate changes in their local regions, identify the symptoms and help them to find the resources to meet their needs [9]. Nurses need to know how climate change affects the care of cancer patients and when it’s a necessary topic to be discussed with patients [15]. Cancer nurses are experts in education and advocacy skills, they can understand and educate the patient that heat waves can increase the risk of heat related diseases and hospitalization [13]. The literature indicates that communication of climate change and its effect on health outcomes can improve shared decision making and its easy to be used by the cancer nurses in their education [7]. Nurses are the largest group of health workers, they have an important role to play in assessing, planning, intervening, and evaluating how climate change affects health, when temperatures are elevated, nurses must check everyone for heat-related illnesses, especially the most frail, patients on chemotherapy treatment and those who are immunocompromised [15]. When the air quality is bad, patients with cancer who may have problems with their heart and lungs should be carefully evaluated; nurses roles also include patient support and advocacy by including cancer related illness with climate change policies, patient education about the role of transportation in decreasing greenhouse emissions, reducing the cancer risk and equally improving survival [12].

Contributing to climate change and its impact on cancer care is the lack of policy in dealing with waste in Palestine. There have been few attempts to sort waste and recycle it. It is thought that only 1% of all solid garbage is being recycled at the moment. In 2010, about a quarter of the waste that was recycled in Palestine was plastic [2]. This number goes up to 3 percent if you count items that have been reused or recycled. Still, recycling and composting have a lot of potential to help solve the problem of increasing amounts of solid waste, as well as to improve cost recovery and create new job possibilities. This is especially true when you consider that most of Palestine's solid garbage is made up of things that can be broken down or recycled. At the moment, recycling and reusing is a small part of the economy in Palestine, and a lot of it is done on the side. It involves recycling plastic, papers, and cardboard. This makes raw materials for local industry, but mostly for businesses in Israel and other places [1]. The major effect of the treatment is to stop harmful compounds from getting into the environment. When these poisons get into surface water and groundwater, they will be very dangerous for people, animals, and plants [3].

## Conclusion

Cancer nursing in Palestine is an evolving profession that is unique in that it faces both political and environmental challenges on a daily basis. With the ever-looming climate crisis impacting upon the global community, cancer nurses in Palestine are ideally placed to advocate and lobby for changes at both a local and national level.

## Conflicts of Interest

Jehad Hawaamdah – Nil, Matthew Fowler regularly volunteers on cancer missions to Gaza and teaches on the MSc Cancer and Palliative Care nursing programme at Bethlehem University

## Funding

The authors did not receive any financial gains in the production of this article.
